# Lifelines and Gateways: The Relational Affordances of Arctic Airports

**DOI:** 10.1177/08912416251398478

**Published:** 2025-12-31

**Authors:** Alexandra Meyer, Ria-Maria Adams, Sophie Elixhauser

**Affiliations:** 1 Department of Social and Cultural Anthropology, University of Vienna, Vienna, Austria; 2 Western Norway Research Institute, Sogndal, Norway; 3 Arctic Centre, University of Lapland, Rovaniemi, Finland

**Keywords:** Arctic, Transport infrastructure, Ethnography, Comparison, Airports

## Abstract

Airports are indispensable to life in the Arctic. Often shaped by geopolitical agendas and external economic interests, they provide vital links for local communities across remote landscapes. However, their societal roles have received limited scholarly attention. By conducting an ethnographic comparison of three cases, this study examines the relational affordances of Arctic airports. Our case studies include Longyearbyen, which is extremely remote but well connected through its airport; Rovaniemi, a central Arctic hub with an international airport and a range of other transport infrastructure; and Tasiilaq, a region that currently does not (yet) have a well-working air connection. While the first towns are established Arctic tourism destinations, the last aspires to increase accessibility and thus tourist visits by establishing a more central airport. The study applies a relational framework that foregrounds the complex socio-material interactions through which infrastructures acquire meaning and function. Our study reveals how Arctic airports simultaneously afford mobility and essential services for residents, enable economic activities such as tourism, and serve broader state and strategic interests. The comparison enables us to capture not only the salient similarities but also the striking differences between Arctic airports according to their local socio-material and environmental contexts, the availability of alternative transport infrastructure, and broader political processes. By centering airports’ relational affordances, this article advances anthropological understandings of Arctic infrastructures and calls for greater local participation in shaping future infrastructural development.

## Introduction

A small plane flies over the iceberg-dotted Greenland Sea towards the island of Kulusuk. Below, mountains and fjords frame the vast inland glacier stretching out like a white ribbon. As the aircraft descends, a cluster of brightly coloured houses appears beside the small airport. East Greenland, home to fewer than three thousand mostly Inuit residents, is both geographically expansive and sparsely populated. It is July 2023, the height of the tourist season, and the cabin is filled with hikers and a few locals. After landing, passengers scatter towards helicopters and small motorboats bound for the small East Greenlandic capital Tasiilaq and nearby settlements.

Some two thousand seven hundred kilometres southeast of Kulusuk lies Rovaniemi Airport, gateway to the capital of Finnish Lapland. Home to about sixty-five thousand eight hundred residents, Rovaniemi is also the ‘official hometown’ of Santa Claus. Each winter, it attracts growing numbers of visitors seeking snowy forests and northern lights. In 2024, nine hundred forty-eight thousand passengers passed through the airport, with one million expected in 2025 (Viinikka 2025). The descent reveals a patchwork of boreal forests, frozen lakes and rivers. Here, winter is less about isolation than orchestration: a carefully staged season where Santa greets arrivals, the conveyor belt glitters with decorations, becoming part of the illusion of a ‘winter wonderland’.

One thousand three hundred forty kilometres north of Rovaniemi, Meyer arrives at Svalbard Airport outside of Longyearbyen just forty minutes before departure. After nearly two years in Longyearbyen, she sometimes takes the risk of arriving late – there’s only one gate, but it can get crowded. Inside, a stuffed polar bear watches over the single conveyor belt, and posters advertise dog sledding, glacier hikes and northern lights. The terminal looks much like any rural Avinor airport on the Norwegian mainland. Meyer greets a miner bound for Tromsø to visit family and a friend travelling for medical care unavailable locally. Tourists queue for souvenirs, scrolling through glacier photos. This ‘northernmost’ town is only a three-hour flight from Oslo – the airport a link that binds distance and proximity in a single gesture.

From Svalbard to East Greenland to Finnish Lapland, airports are indispensable to life in the Arctic. While transport infrastructures in the Arctic have often been shaped by geopolitical agendas and external economic interests, airports, in particular, serve local communities as vital links across remote landscapes. Arctic airports serve multiple functions, consolidating strategic interests, enabling extractive industries, facilitating tourism flows and supporting daily life. They are essential for the movement of residents, tourists, fly-in-fly-out workers, military personnel, scientists and other visitors.

The Arctic’s low population density and dispersed settlements create persistent challenges for connectivity and economic activity (Gløersen et al. 2009). Long distances, harsh weather, rugged terrain, limited road and rail infrastructure, and seasonally restricted sea access make airports not only central to accessibility but also lifelines for many communities. Today, the Arctic hosts around one thousand three hundred airports and heliports – including seven large and two hundred sixty medium-sized airports with regular passenger traffic (Nordregio 2019). Yet, despite their importance, the societal roles of Arctic airports have remained largely overlooked in social science, ethnographic research to date being limited.

This article seeks to rectify this gap by extending discussions on specific local case studies to inform broader debates on the role and affordances of Arctic airports. By comparing the multiple affordances of three Arctic airports – Longyearbyen (Svalbard), Kulusuk (East Greenland) and Rovaniemi (Finnish Lapland) – we present an empirical contribution to the growing literature on Arctic transport infrastructure and the broader anthropology of infrastructure. The sites differ substantially in structure, economy and availability of other transport infrastructures, but share marked similarities in being imagined by the growing tourism industry as remote Arctic wilderness destinations.

We set out to address the overarching research question: *What do Arctic airports afford local communities and their residents?* In addressing this question, we contribute to anthropological discussions of transport infrastructure. We examine the question in terms of relational affordances (Gibson 1979; Ingold 2000, 2022), which directs attention to the possibilities airports offer the various actors – residents, industry and states – that engage with them. This view considers both the materiality of the infrastructure as such and the perceptions of those using and affected by it, allowing us to think about the effects and functions of infrastructures without resorting to technological determinism. Furthermore, the perspective chosen directs our attention to the infrastructural relations (Buier 2023) whereby airports offer affordances such as connectivity and mobility.

## Relational Affordances of Arctic Airports

We situate our discussions in the expanding field of the anthropology of infrastructure (Anand et al. 2018; Buier 2023; Harvey et al. 2017; Kanoi et al. 2022; Larkin 2013; Star 1999) and, in particular, the anthropology of transport infrastructure. Notwithstanding the diversity of approaches to infrastructure, some scholars see the potential of infrastructure as an analytical lens that specifically redirects our attention towards socio-materiality and relationality (Buier 2023). In the light of Buier’s work in particular, we have chosen a focus on relationality, as this allows us to study infrastructure assemblages (Taufen et al. 2022) and continuous complex interactions between the socio-material actors and processes. Relationality lies at the heart of the affordance perspective, introduced by ecological psychologist Gibson (1979) and later applied in anthropology by Ingold (2000, 2022) to analyze human–environment relations. To Gibson (1979), ‘[t]he *affordances* of the environment are what it *offers* the animal, what it *provides* or *furnishes*, either for good or ill’ (127). Affordances are relational, depending equally on the environment and the perceptions and actions of the animal (including humans). Applied to the study of infrastructure, this approach emphasizes that affordances are not fixed or inherent properties of a particular infrastructure, but rather emerge through interactions between actors and the materiality of the infrastructure within specific contexts. Infrastructural affordances are thus co-constituted through practice and shaped by socio-material relations, through which infrastructures acquire their significance. This relational view shifts the focus from the infrastructure itself to its effects and its functions, which in turn depend on the perceptions, intentions and actions of those who interact with it. A focus on materiality has been criticized for relying on a notion of matter as something pre-social (Buier 2023, 51), thus detracting from social relations, power and politics (Hornborg 2018). Taking this criticism into account, the concept of affordances allows us to study airports without letting the infrastructure itself ‘get in the way’ of what we want to study. It directs our attention to the (infrastructural) relations and the socio-material processes at work, emphasizing that the social and the material spheres are inextricably linked and mutually constitutive (Reeves 2017). A relational perspective furthermore suggests that ‘remoteness’ cannot be reduced to spatial dimensions and equated with marginality (Ardener 2012). Rather, it is always deeply enmeshed in the historical, political and social conditions of a particular region and is actively created, undone or re-created (Saxer and Andersson 2019).

Transport infrastructure links outside and local interests, shapes mobility patterns and enables resource extraction. A central affordance of transportation infrastructure is to overcome remoteness through connectivity (Harvey 1990). Following Saxer and Schorch (2020), connectivity is not merely understood as a connection between discrete entities, but as a mode of *becoming*. As they contend, ‘[j]ust as things tend to become the things they are through the connections in which they are engaged, connections are often imbued with material qualities’ (Saxer and Schorch 2020, 2). In the Arctic, efforts are underway to enhance connectivity within the region, between peripheral areas and global networks; this work is often guided by imaginaries of progress such as business development and increased services (Abildgaard et al. 2022). However, spatial distance might be perceived as a resource in certain contexts (Schweitzer and Povoroznyuk 2019), which problematizes notions of connectivity underpinned by the logic of ‘the more, the better’ (Abildgaard et al. 2022). Anthropological engagements with transport infrastructure have focused on roads (Clarke 2020; Dalakoglou and Harvey 2012) and demonstrated their potential to both connect and disconnect in reinforcing political and material orders. Railroads and seaports have been studied as agents of social change and modernization (Anand et al. 2018). Airports, although critical infrastructure at the heart of globalization, have received less attention in the field. They have been approached as non-spaces (Augé 1995) and transit zones characterized by formality and control (Chalfin 2008). Some scholars contend that they create particular subjectivities as people become passengers (Adey and Merriman 2012; Shilon and Shamir 2016) and possibly reinforce hierarchies (Aaltola 2005). Others have studied the experiences and practices of passengers, including practices of waiting (Schindler 2020). Arctic transport infrastructures were often built to satisfy outside interests, and their design is characterized by ‘their non-local origins and intentions’ (Schweitzer 2020). Air transport is of particular relevance in remote and sparsely populated areas like the Arctic (Schweitzer 2023), where airports and aviation have played crucial roles in exploration and colonization (Arctic Aviation 1928; Cronin 2010).

What remains less researched is the role of the region’s airports for the residents that rely on them. Recent ethnographic research on Arctic airports has examined topics spanning the unpredictability of air travel and passenger waiting (Davydova 2024), hopes and socio-economic challenges (Elixhauser 2023), affective investments surrounding new airports (Ren 2023; Vanek forthcoming) as well as tourism development and connectivity (Ren et al. 2024). The work has also broached broader economic and geopolitical framings, including cost–benefit analyses and securitization (Christensen et al. 2020; Sejersen 2024). Most studies of Arctic airports focus on Greenland, where several facilities are under development. This study broadens the scope by including and contrasting one novel site in Greenland and two elsewhere in the Arctic.

## Methodology

Comparison has been critiqued for its potential to flatten cultural specificity (Abramson and Gong 2020; Niewöhner and Scheffer 2010). We align with more recent approaches that foreground *thick comparison* (Niewöhner and Scheffer 2010) as a way to remain attentive to the partial, situated and processual nature of the phenomena under study. Rather than seeking universal categories or predetermined axes of difference, our comparison traces what Arctic airports afford different actors. Fundamentally, we argue that comparison is not a mode of simplification, but a means to render visible the multiplicity of forms that infrastructures, and their surrounding lifeworlds, can take across contexts.

The present research is based on a comparison that was conducted as part of the InfraNorth project from February 2022 to August 2024. Our framework is guided by a problem-centred analysis that extends beyond simply contrasting individual case specifics. We seek to uncover the broader abstract entity ‘Arctic airport’ as a shared infrastructural entity whose affordances, functions, impacts and significance can be generalized to other Arctic contexts. The comparison thus operates on two levels: it puts forward bottom-up, ethnographic insights and provides a meta-level synthesis that identifies cross-cutting themes and patterns.

In all three regions, the study on the airport was woven into long-term ethnographic research engaged in a variety of topics. In Longyearbyen and Rovaniemi, fieldwork took place between 2018 and 2024; in Greenland fieldwork started already in 2005 and came to include a specific study on the topic of airports in 2023. The main methods for data collection encompassed unstructured and serendipity-driven participant observation, informal conversations and structured interviews. In each location, our sampling approach combined random snowball sampling with purposeful sampling (Lareau 2021). We interviewed airport personnel and management, local politicians and administrators, tourist companies, business owners, as well as a wide range of residents of all ages and genders from various socio-economic, ethnic and national backgrounds.

For the comparison, each researcher constructed inductive categories from their data, which we then merged with existing categories from previously analyzed material. The two sets of data were then collaboratively compared, with categories being further refined with regard to the research question and theory. The first phase of the analytical process included inductive and deductive elements as well as individual and collaborative phases, and the research design included both a priori and a posteriori comparative perspectives. The second main element of the methodology then comprised explicit qualitative comparison (Palmberger and Gingrich 2003). The main categories that emerged from this comparison were strategic and military affordances; affordances for local communities (mobility of residents for leisure, work, healthcare; cargo transport; emergency and preparedness); isolation and connections to other transport infrastructure; and the local and supra-local dimensions of tourism affordances.

## From Geopolitics to Everyday Life and Tourism: The Multiple Affordances of Arctic Airports

Although most Arctic airports were established either for military purposes or regional development (Halpern and Niskala 2008), their roles have since diversified. We now outline the strategic significance of the three airports studied before turning to two key affordances: first, how airports meet local needs, and second, how they support tourism demand.

The influence of military and geopolitical interests on these sites varies across cases. Rovaniemi Airport serves both civilian and military functions; its military role has expanded since Finland joined NATO in 2023, bringing changes not only to airport operations but also to local communities, particularly Indigenous Sámi reindeer herders, who must contend with both spatial restrictions and increased noise from military exercises (Junka-Aikio 2024). Military exercises have increased, and infrastructure is being developed to support these activities. By contrast, Svalbard Airport is a strictly civilian facility due to treaty restrictions prohibiting military activity. Yet it remains strategically important, enabling Norway to maintain a permanent settlement and thus uphold sovereignty in the region. Kulusuk Airport was originally built for Cold War radar surveillance but has since transitioned to civilian use. Nevertheless, its remote location reflects strategic rather than local priorities, and although military activity is currently minimal, geopolitical interest in Greenland persists. Together, our three airports demonstrate how Arctic airports are not merely infrastructure but also enduring strategic assets embedded in global entanglements.

### Everyday Airport Affordances for Residents

Like many of Greenland’s larger and older airports, Kulusuk Airport was originally built during the Cold War for military purposes (Ancker 1999; Elixhauser 2018). Passengers travelling from Kulusuk Airport to the small East Greenlandic capital Tasiilaq (pop. 1,800) take a ten-minute helicopter flight, and the Tasiilaq heliport provides helicopter connections to the other regional villages. This combination of planes and helicopters often results in delays or cancellations due to weather conditions, availability or technical issues. Kulusuk Airport is connected to Nuuk, the capital, by Air Greenland flights. Icelandair also offers regular services to and from Keflavik International Airport. However, this route is becoming increasingly restricted, particularly during the winter months. Air tickets in Greenland are generally expensive, though there is some hope that this is about to change. Air traffic is vital for work, education and leisure travel as well as for transporting patients in emergencies, and for search and rescue operations. In East Greenland, helicopter availability is often an issue, with passengers sometimes left stranded. Moreover, Air Greenland is often criticized for prioritizing flights to West Greenland over those to East Greenland. Tasiilaq residents told Elixhauser that the latter are cancelled much more often. Nevertheless, the infrastructure at Kulusuk Airport and the nearby village is not geared to hosting passengers who may not be able to proceed as planned.

Due to the difficulties in air travel, plans for a new and more central airport in Tasiilaq have been debated for over thirty years. One major hope of the Greenlandic government is that an airport in Tasiilaq will generate new revenue through tourism, income needed to fund the aspired future independence of Greenland (Hall 2020). However, for Tasiilaq’s inhabitants, a new airport would mean much more, as Elixhauser’s field research showed. Various socio-economic hopes feature centre-stage, related to economic opportunities as well as a wish to reduce the perceived remoteness of the region and concomitant feelings of exclusion and powerlessness. Tasiilaq, which features regularly in the Greenlandic press due to a high number of suicides, alcohol problems and other social issues, is located far from all the country’s seats of power; for example, the municipal centre, Nuuk, is located on the opposite coast. This physical distance (six hundred forty-eight kilometres Tasiilaq – Nuuk) and the challenges of access, combined with negative media coverage and various prejudices throughout Greenland about the East Coast, have led to a widespread sense of disconnection, both social and physical, among the East Greenlandic population (Elixhauser 2023). Some residents hope that a new airport would provide a new positive self-image, ultimately helping to combat the prevailing social problems. This aspiration has been captured by Justus Hansen, a Tasiilaq politician, member of the Greenlandic parliament and passionate advocate of a new airport, who stated in an interview: ‘We want to be proud, a place, not to be the ‘back side’. To have a positive place in the media, not only [stories] about suicide, raping, child abuse, etc.’

Greenland’s airspace is currently undergoing major restructuring. Three new airports have been built or are under construction in central towns on the West Coast of the island (Christensen et al. 2020). Nuuk has expanded to become an international airport, which opened in 2024. At the time of writing this article, the new international airport in Ilulissat was nearing completion, with an expected opening date of late 2026. Construction of a smaller regional airport in Qaqortoq in southern Greenland is also expected to finish around this time. Initially included in Greenland’s 2015 coalition agreement alongside the three aforementioned airport projects, the new airports for Tasiilaq and Ittoqqortoormiit, the only two towns on Greenland’s sparsely populated East Coast, were dropped in 2017, much to the disappointment of many residents. A 2017 Sermitsiaq newspaper article, for example, was titled: ‘It is so incredibly disappointing that we on the East Coast are being forgotten again and again. It kills people’s dreams’ (Düwel 2017). Thus, the issue is framed as part of the wider issue of the perceived neglect and disenfranchisement experienced by residents of East Greenland within the nation-building process (Elixhauser 2023). Following the elections in Greenland in April 2025, the construction of a new airport in Tasiilaq (as well as Ittoqqortoormit) was again listed as a priority. However, financial constraints have once again put the project on the back burner.

The increasing sense of neglect experienced by East Greenlandic residents was voiced during a demonstration in Tasiilaq on 18 May 2025; more than 600 people walked through the town carrying banners reading ‘Enough is enough’, ‘Silenced’ and ‘Dunu [East Greenland] lives matter’. Co-organizer Iddimanngiiu Jensen Bianco told Greenlandic broadcaster KNR that the reasons for the demonstration included poor travel conditions, poor internet connectivity, a shortage of teachers, inadequate housing and unemployment. ‘These are the consequences of being put at the back of the queue. There is no development in the city’ (Møller et al. 2025; authors’ translation). As Mike Nicolajsen, a businessperson and hotel owner, told KNR, ‘Enough is enough. We don’t want dismantling; we want development in East Greenland. We need to make it clear that we East Greenlanders exist, too’ (Møller et al. 2025).

**Figure 1.**
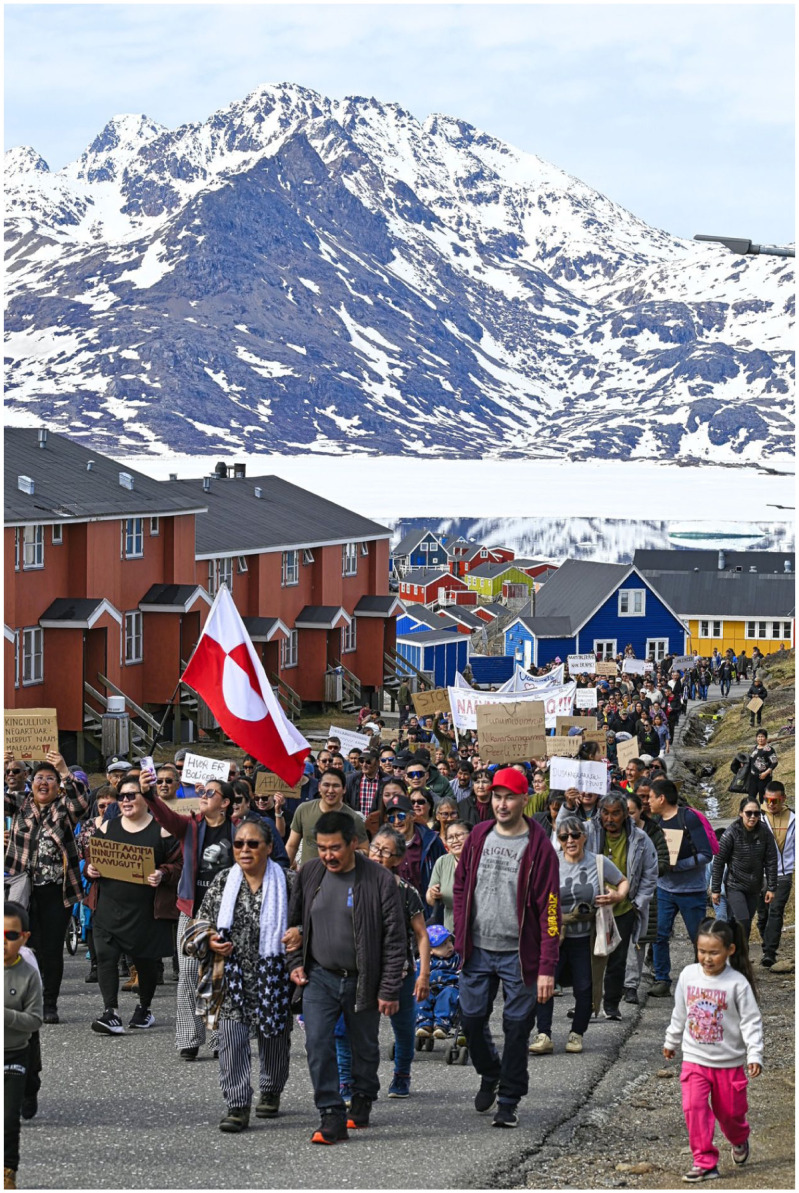
Demonstration in Tasiilaq, 18 May 2025. Photo by Alex G. Hansen.

The demonstration was partly organized in response to Icelandair’s decision to temporarily suspend the flight connection between Reykjavik and Kulusuk from October 2024 to March 2025 (Schultz-Nielsen 2025). This has led to much frustration in Tasiilaq, a town which is experiencing population decline, as it forced some residents to take costly detours via Nuuk when travelling to or from Denmark, Iceland and other countries. More broadly, it endangers the goods, know-how and trade relations with Iceland that Tasiilaq has relied on for decades. In the demonstration, local tourism entrepreneurs expressed general fears for the future of tourism in East Greenland and for the future of Tasiilaq. When Naalakkersuisut, the Government of Greenland, failed to respond to the demonstrators’ demands, a new political party – the Party for an Independent East Greenland – was founded by some actors in Tasiilaq in July 2025, calling for a complete break from Greenland after years of accumulated frustration (Figure 1; Hansen Balle 2025).

Longyearbyen is the largest settlement and administrative centre on the Svalbard archipelago, located about midway between the Norwegian mainland and the North Pole. Svalbard never had an Indigenous population and was considered a *terra nullius* until the Svalbard Treaty, which entered into force in 1925 and granted Norway sovereignty. The Treaty states that Svalbard is a demilitarized zone and grants all citizens and commercial entities from the signatory states equal access to and the right to engage in commercial activities on the islands. Once a coal-mining town, Longyearbyen (pop. 2,600) is today a highly transient and international community, with an economy centred on tourism, research, education and state subsidies (Sokolíčková et al. 2022). Transience shapes local social life, as many residents maintain multiple attachments to several places – a lifestyle enabled by the connectivity afforded by the airport. Svalbard holds strategic significance for Norway, with the maintenance of a permanent settlement seen as essential for asserting national sovereignty. This broader political objective shapes everyday life in Longyearbyen, including operations at the airport. In a 2023 interview, the airport manager emphasized that Svalbard Airport’s development is guided by Norway’s overarching Svalbard policy. Here, the drive for growth is moderated by strategic considerations, including the regulation of tourism and infrastructure expansion. With Russia present on the archipelago, infrastructural developments on Svalbard need to be carefully weighed in terms of their potential geopolitical ramifications. This is also the reason for the relatively late construction of an airport in Longyearbyen (Tamnes 1992).

As part of its efforts to transform what was a company town into a ‘normal’ family community (Arlov 2003), the Norwegian state advanced its plans to construct an airport in Longyearbyen in the 1970s, aiming to reduce its isolation by offering regular, affordable direct flights to the mainland. After diplomatic negotiations, Russia agreed to a civilian airport with limited Russian presence, and Svalbard Airport Longyearbyen opened in 1975 (Tamnes 1992). Until then, Longyearbyen had relied on an unpaved runway on the frozen tundra, operational only during the winter months. This limited air access effectively isolated the town in winter (Solberg 2019), as sea ice and darkness prevented boat traffic. While some longtime residents recall this period with nostalgia (Meyer, forthcoming), those whom Meyer interviewed agreed that year-round access to the mainland is essential to Longyearbyen’s current way of life. Thanks to its modern infrastructure and strong air links to mainland Norway, Longyearbyen today feels more like a typical Northern Scandinavian town than a remote Arctic outpost. The airport offers the primary means of connectivity with the mainland (the journey by boat to or from the Norwegian mainland takes at last two days and is marketed as a tourist experience, with prices to match). Residents thus rely on the airport to access comprehensive healthcare on the mainland, as the facilities and services of Longyearbyen hospital are limited. With frequent, affordable, and fast connections to Oslo and Tromsø, residents regularly fly ‘down’ for business meetings or to visit family, much as they might make such trips by car, bus or train on the mainland. Here, the airport enables a particular sociality, allowing people in what is a transient and highly mobile community to cultivate multiple sites of belongings, although living in the supposedly remote high Arctic. This connectivity furthermore supports a thriving tourism industry and positions the town as a central hub for Arctic research and education. In an interview, airport personnel noted that while disruptions due to bad weather and visibility occur, they rarely affect Longyearbyen’s connectivity for longer periods. However, they explained, climate change presents the bigger challenge, leading to permafrost thaw underneath the runway, increased water run-off from the nearby slopes and changing wind directions.

**Figure 2.**
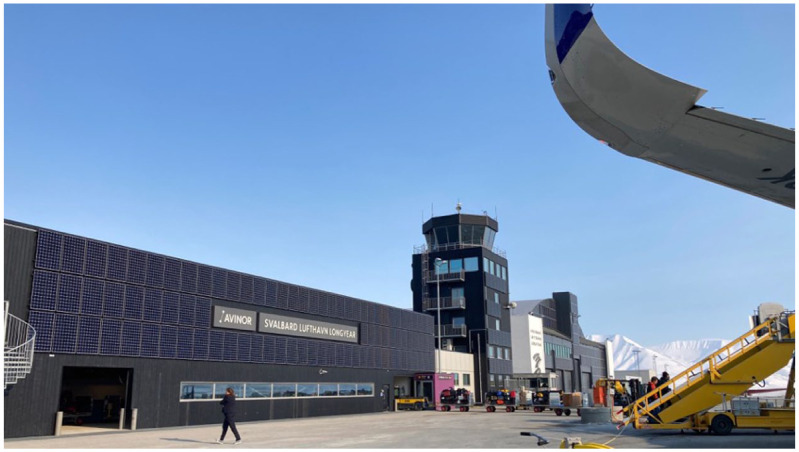
Svalbard Airport Longyearbyen. Photo by Alexandra Meyer.

Svalbard Airport, owned by the state-owned company Avinor, is today operated as part of and integrated into Norway’s broader network of domestic airports. The schedule includes direct flights daily to Tromsø and Oslo at reasonable prices throughout the year. In addition, the airport affords passenger transportation to the Ny-Ålesund research station and various missions. The Russian mining company Trust Arctic Kugol shuttles employees and their families between the airport and the mining settlement Barentsburg by helicopter, and the airport also facilitates charter flights, notably for expedition cruise ships. For patient transport in cases of emergency, Longyearbyen is dependent on two long-range rescue helicopters that have the capacity to fly to the mainland (with a stopover on Bjørnøya island). Due to Svalbard’s remoteness, the air ambulance constitutes a ‘vital service’ and, as the recently published white paper for Svalbard states (Meld. St. 26 2023–2024), the new helicopter hangar at Svalbard airport represents a significant increase in emergency preparedness and safety for residents and tourists alike. Longyearbyen is accessible year-round by waterway, and most goods are transported by ship. Yet, many essential goods, such as fresh produce for the supermarket, medicines for the hospital and pharmacy and mail are transported by plane, both on passenger flights and on the bi-weekly mail airplane operated by the Norwegian mail service Bring. In 2025, Bring transitioned to scheduled flights and maritime transport for mail and goods, citing economic and environmental considerations. This decision has sparked concerns about food security and emergency preparedness by local business operators (Bjørkhaug 2024). In an interview, the airport manager described the airport as a *crucial pillar of Longyearbyen society o*wing to its multiple affordances. However, she also noted that this dependence also renders the community vulnerable, emphasizing that ‘we feel a very big responsibility to keep the airport open’ (Figure 2).

Our third case, Rovaniemi Airport, offers an important and compelling point of comparison due to its Arctic location on the mainland of northern Europe and its integration into a broader network of transport infrastructures beyond air travel. Unlike the previous two cases, where airports serve as vital lifelines for services such as food delivery, healthcare, and mail, as well as enabling travel to and from the communities, Rovaniemi’s airport plays a less existential role for the local population. This contrast makes Rovaniemi a valuable example for examining the varied local affordances of Arctic airports. As the capital of Finnish Lapland, Rovaniemi functions as a regional hub for the more northern and remote communities in the area. It offers essential services such as a central hospital, national and local administrative offices, educational opportunities (including a university) as well as shopping and recreational opportunities. Unlike in Greenland or Svalbard, where helicopters are a common mode of transport from isolated settlements, residents of northern Finland typically travel to Rovaniemi by car because of the well-developed road network. This highlights a key difference in how mobility and access are structured across Arctic regions and further underscores the varying roles airports play in meeting local needs. What further distinguishes Rovaniemi is the airport’s military function, which has gained renewed significance since Finland joined NATO in 2023. Historically, the airport was used for military purposes, and its strategic role continues to shape both its operations and its relevance within broader geopolitical developments in the Arctic region.

**Figure 3.**
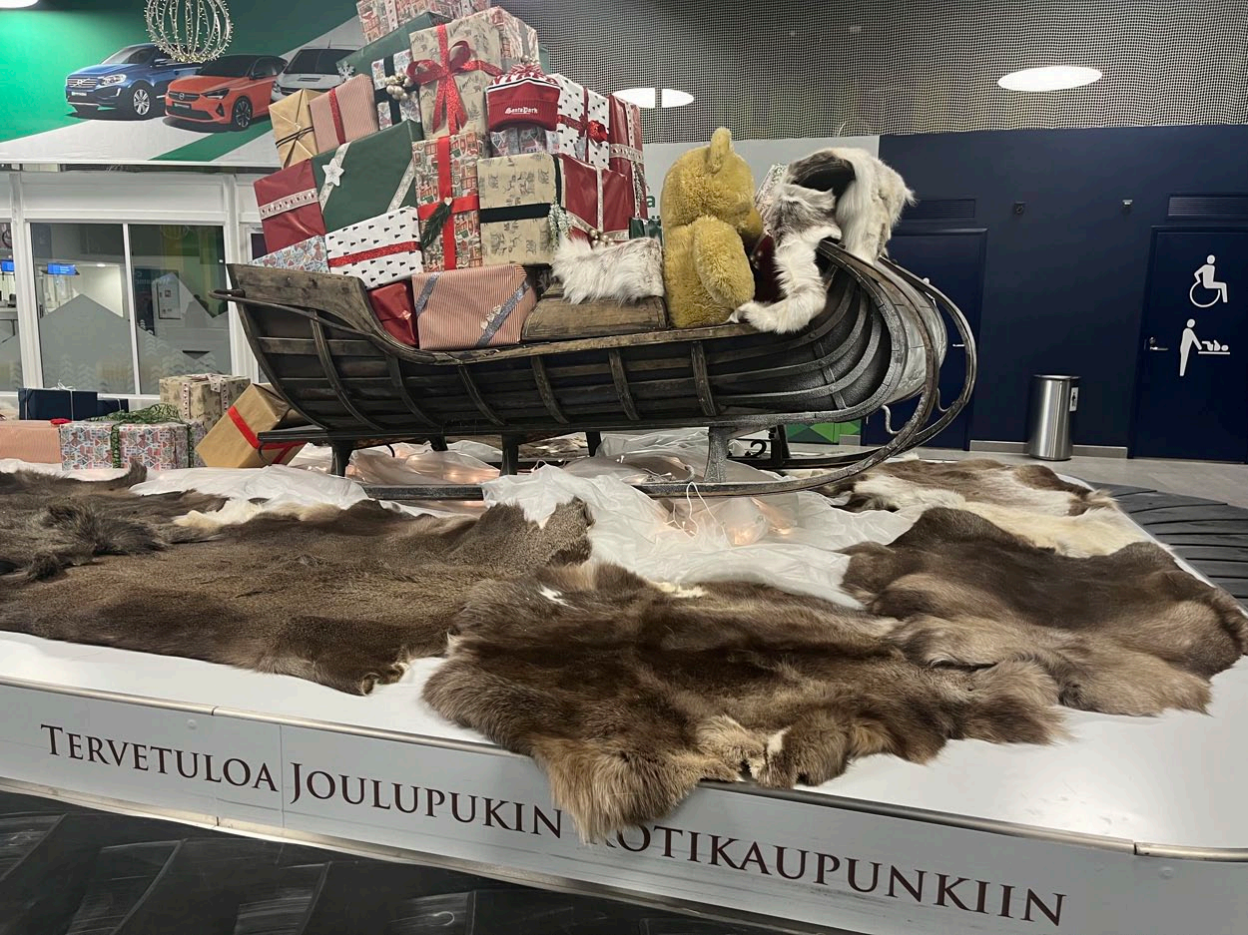
Christmas-decorated conveyor belt at Rovaniemi Airport, November 2023. Photo by Ria-Maria Adams.

Construction of the Rovaniemi airport started in 1939 (Finavia 2025). When the Continuation War began in 1941, the recently completed facility served as a military base for the German armed forces and was significantly expanded. At the end of the war, the Germans destroyed the airport, leaving nothing but ashes along with mines, bombs and ammunition that had to be cleared (Mikkonen 2022). In 1948, reconstruction began on the site, marking the start of its postwar recovery. In 1974, the Lapland Air Command started using the facility as an airbase. Rovaniemi Airport, like most airports in Finland, is operated by Finavia Corporation – a fully state-owned public company (Finavia 2025). This makes the airport similar in structure and service standards to other Finavia-operated airports across the country, offering a consistent and familiar experience, much like the standardized arrangements observed in Longyearbyen. The national carrier Finnair operates several daily flights between Helsinki and Rovaniemi throughout the year for mostly reasonable and affordable prices and during the summer months may at times be the main and only operator. In winter, an increasing number of low-cost airlines compete with Finnair for passengers and the 2024/2025 season saw an all-time high of forty-one direct flight connections from various European cities (Visit Rovaniemi 2025). The current airport terminal in Rovaniemi was expanded in 2019 to accommodate growing passenger demand. However, during the peak Christmas travel days of the 2024/2025 winter season, reports of passengers waiting for up to an hour in freezing temperatures just to enter the building have prompted further investment. In response, the terminal is undergoing another expansion, with a €3 million upgrade to improve capacity slated to be completed in time for the 2025/2026 winter season (Finavia 2025). In 2024, the number of passengers at Rovaniemi Airport increased by nearly 30 percent compared to the previous year, reaching 948 000 passengers, many of them international visitors. However, when considering the local affordances of Rovaniemi Airport, statistics from 2024 show that domestic travellers still accounted for 56 percent of all passengers, while international flights made up the remaining 44 percent (Finavia 2025). Although a majority of flights are domestic, the airport’s year-round Christmas decorations are clearly geared towards tourists rather than local residents. In Rovaniemi, the airport operates exclusively as a passenger hub, with no goods being transported through it. In an interview with Adams, the airport manager explained that developing facilities for freight traffic would require additional and different logistics, including a distribution centre. Although consultants have previously considered adding a freight hub to the airport, it is currently not feasible. At present, transporting cargo by road and rail appears to be more practical, as logistics centres for the purpose are already well established. The airport is one among many elements of the city’s infrastructure that facilitate mobility for the population. While the airport plays an important role for residents of Lapland, particularly for those with family or work commitments in the south, the airport would have far fewer passengers without tourists and the large-scale investments now planned would not take place. Despite its Arctic location, Rovaniemi’s airport is not particularly vulnerable to weather conditions. Disruptions are rare, but the town has sufficient hotel capacity to accommodate any stranded passengers should a flight be cancelled. This stands in stark contrast to more remote locations such as Tasiilaq (Figure 3).

### Tourism Affordances

Tourism plays a central role in all three towns and relies heavily on the presence and functioning of their airports. Rovaniemi stands out as the primary tourism hub, with passenger numbers projected to exceed one million in 2025 (Viinikka 2025). Airports both enable the flow of visitors and derive their own viability and expansion from the rhythms and demands of the tourism industry. In this reciprocal relationship, the existence, accessibility and seasonal dynamics of air travel fundamentally shape what forms of tourism are possible, desirable and sustainable (Sinanan et al. 2025).

Between July and December 2024, registered overnight stays in Rovaniemi grew by 20 percent compared to the same period the previous year, while unregistered stays, including those booked through platforms like Airbnb, rose by 48 percent (Viinikka 2025). This growth underscores the importance of tourism to Rovaniemi’s economy, which relies heavily on visitor spending to support employment and local services. At the same time, the sector’s rapid expansion is reshaping local infrastructures. The surge in short-term rentals has sparked some local resistance, although protests remain small-scale, as many residents also benefit from the additional income generated by tourism. One resident described how their family of four packs all their belongings into their garage for a few weeks every December to rent out their entire house. Short-term rentals often generate more income than traditional 12-month leases at standard rates, exacerbating the housing shortage for permanent residents. The core issue lies in the seasonal demand: short-term rentals see high occupancy from November to March, but many apartments remain vacant for much of the rest of the year. During the high season, tourists struggle more with securing suitable accommodation than with flight capacity, making lodging challenging even when there are seats available on flights.

At a local demonstration in early September 2024, Adams observed residents marching through the city centre carrying signs that read: ‘My home is not your hotel’, ‘Laws need to be followed’, and ‘Apartments for residents. Hotels for tourists’. While these protests do not directly mention the airport, the connection between the growing influx of tourists and the pressures on local housing – and, by extension, local affordances – is unmistakably linked to the flow of visitors arriving through Rovaniemi’s transport infrastructure. Other infrastructures, such as roads, also become strained during peak holiday seasons, as tourists who rent cars and are unfamiliar with winter conditions often drive off the road or onto ski tracks and walking paths – causing frustration among locals (Adams and Bennett 2026). Tourists walking on main roads, crossing at unsafe spots, walking on thin ice on rivers and lakes, or misusing public amenities like outdoor campfire sites further contribute to safety concerns and growing debates about the misuse of local infrastructure. All of this is enabled and afforded by the airport, which serves as the primary gateway bringing large numbers of visitors into the region.

In an interview, the manager of Rovaniemi Airport noted that the number of regular flights was expected to continue rising, with private aircraft traffic steadily increasing as well. The airport and its carriers have occasionally encountered unusual requests. For instance, as the airport manager recalled, a Finnair aircraft scheduled for an overnight stay at Rovaniemi Airport was chartered by a Japanese tourist group, with only the window seats available, for a special one-hour flight above the clouds to view the northern lights. Although such practices are not commonplace, they highlight the tourism industry’s creativity in accommodating the desires of more wealthy customers.

In Longyearbyen as well, tourism has grown to become the main economic sector and is highly dependent on the airport. Approximately in 2024. 60% of all full-time equivalents in Longyearbyen were related to tourism (Salte Flatval et al. 2025), and tourists arrive almost exclusively by plane and cruise ship. The contribution of cruise passengers to the local economy is limited, as they sleep and consume services on the ships. In contrast, the tourists flying to and staying in Longyearbyen, or using the town as point of departure for expedition cruises, play an essential role in the town’s economy (Saville 2022). In 2017, the status of Svalbard airport changed from international to national, and direct routes from outside of Norway were no longer permitted. Officials cited logistical reasons for this change of status, stating that the size of the airport does not permit the security controls required of an international airport (Finne 2017). Local business representatives claimed that several international airlines had expressed interest in establishing direct routes to Svalbard, and objected that the change meant a serious setback to tourism development (Røsvik 2017). Nevertheless, tourism has continued to grow and in 2024, 67 656 “guests” arrived in Longyearbyen (Visit Svalbard 2025).

Locally, there are also critical voices questioning the growth of tourism, highlighting precarious working conditions, a housing shortage, and local and global environmental impacts from an industry heavily reliant on aviation and cruise ships. In an interview, a community planner expressed her frustration to Meyer: ‘We’re trying to make this place more sustainable, taking all these local measures, but sometimes I wonder – what’s the point, if our economy depends on people flying up from the South [. . .] to see the glaciers before they disappear?’

In recent years, the Norwegian government has put forward a non-growth policy for Longyearbyen, increased state control over urban and community development, and tightened environmental regulations. The Svalbard airport has to navigate the resulting tension between economic interests and development on the one hand, and state control and increasing regulations on the other, a dilemma the airport manager expressed in the following way:

At all other airports, it is, in many ways, demand and the market that determine how you should further develop an airport. And then you have Svalbard. Here there are completely different factors that seem to impose much stronger constraints on development opportunities than what market needs or demands would suggest.

The new airport projects along Greenland’s West Coast, including new air links such as the connection between Nuuk and New York, which opened in the summer of 2025, are intended to tap into new tourism markets and capitalize on the expected growth in tourism. The new tourism strategy for Greenland, which runs from 2025 to 2035, focuses on sustainable and community-based development. However, tourism in East Greenland is facing the opposite challenge due to the aforementioned transport infrastructure problems. Accordingly, most local tour operators are in favour of a new airport in Tasiilaq and are keen to see growth. As of 2024, East Greenland featured twenty thousand seven overnight stays (Statistics Greenland 2025). Tourism is highly seasonal, taking place primarily in the summer months with a smaller peak in spring (Statistics Greenland 2025). Similarly to Svalbard, there is also cruise tourism, although it is less relevant to the local economy. In an email exchange (2023), a local tour operator emphasized that a new central airport would be crucial for bringing ‘people directly into Tasiilaq’, as it would eliminate the current delays caused by reliance on helicopters or boats. The general tone in the region is one of support for the growth of tourism as an important economic opportunity, underpinned by the hope that it could help alleviate social problems. Yet, there are voices of caution pointing out possible negative effects of a growing tourist industry. one of the hotel owners in Tasiilaq, for example, is supporting a new airport in the town but one with only a small landing strip. This would prevent the area from being flooded with travellers. When Elixhauser spoke with some residents of the village Kulusuk about their opinions on a possible new, central airport in Tasiilaq, many were critical. They were afraid that the closure of Kulusuk Airport would deprive the village of its livelihood by taking away jobs in and around the current airport and bringing an end to the important one-day tourism from Iceland. One tour agent reacted quite intensely: ‘Not in my lifetime. [. . .] It would destroy the whole community here. People would slowly move away, and not much would stay’. An article by Markussen and Ren (2023) draws a similar critical view of the wide-spread wish for growth in tourism in Tasiilaq. Interviews with key stakeholders in Tasiilaq revealed several structural challenges for tourism growth, such as a lack of trained tourism staff. Residents emphasized their desire for culturally appropriate tourism and local ownership – a desire frequently discussed by stakeholders and residents in all our case sites.

## Discussion and Concluding Remarks

Taking a relational affordance-based approach to examining Arctic airports shows that transport infrastructures and their societal effects differ vastly across specific socio-material contexts. Although all three airports – Tasiilaq/Kulusuk, Rovaniemi, and Svalbard – offer air connectivity, their affordances differ, depending not only on their materiality but also on the actions and intentions of those who interact with them, and their socio-environmental contexts. This highlights the inherently relational nature of transport infrastructure and the intricate interplay of socio-material processes and assemblages that determine their significance for different actors as well as the expectations associated with them. Importantly, the affordances of the three airports for residents are determined by the presence or absence of other infrastructure and alternative modes of transport. On Svalbard, a remote archipelago without fixed links to the mainland, the airport offers quick and affordable connections to the outside world, making it essential for the day-to-day functioning of the community. In East Greenland, the lack of roads between settlements means that airports play a similarly important role. However, the weather and the reliance on a combination of airplanes and helicopters to reach the regional centre of Tasiilaq impose constraints on connectivity. While Longyearbyen and East Greenland are accessible only by air and sea, Rovaniemi in contrast benefits from alternative transport infrastructure connections, in particular well-developed rail and road networks which ensure that people can still access and leave the town via multiple routes. This availability of transport alternatives makes the airport less vulnerable to non-human entities and processes such as a thawing ground, wind, weather and a changing climate, which affect connectivity in East Greenland and Longyearbyen. This underscores the deeply relational nature of transport infrastructure (Buier 2023), revealing how its affordances are shaped by their integration within wider, multimodal networks, including social, material and non-human processes.

Arctic transport infrastructure has often been shaped by external interests. Our comparison shows that while Arctic airports continue to serve outside demands – such as strategic priorities and tourist’s pursuit of Arctic remoteness – they provide essential local affordances for Arctic residents, albeit ones varying across the cases. In Longyearbyen and Tasiilaq, they serve as vital lifelines: they are crucial for passenger transport for work, education, leisure and healthcare; cargo deliveries and economic activity; and search and rescue operations. In Rovaniemi, while the airport might appear less central to residents’ daily lives, it plays a key role in sustaining a booming tourism industry, now a cornerstone of the local economy.

Across all three locations, the airports afford essential connectivity for a growing tourist industry, highlighting both the sector’s growing influence in the Arctic and its dependence on air travel. The affordances the airports offer tourism are at the same time local and global in scale: in all three locations, the tourism industry is central to the local economy, while also enabling non-local companies (such as airlines and tourist operators) to profit. The airports afford connectivity to tourists, acting as gateways that make these ‘remote’ destinations, which are often marketed as ‘pristine’ Arctic landscapes (Klinger et al. 2023), accessible. This creates a paradox in which the very remoteness being commodified is at risk of erosion through the processes of tourism development (see also Ren et al. 2024). Indeed, connectivity is not only a logistical necessity but also a prerequisite for the commodification of Arctic remoteness itself. In all cases, tourism, facilitated by air transport, comes with advantages in terms of economic growth and jobs and concerns such as environmental degradation and societal challenges. Hence, improved connectivity through a new airport (East Greenland), international routes (Svalbard) or more flights (Rovaniemi) is controversial in all three cases. While the needs of inhabitants have been the focus for recent developments, the increasing geopolitical tensions in the Arctic suggests that military and strategic interests might dictate future developments to an even greater degree than they have to date.

The comparative approach taken here has broadened our understanding of the role airports play in the Arctic while revealing the nuanced ways in which they function within their specific contexts. Although some affordances may remain unobserved, with ‘the Arctic airport’ remaining an abstraction, the comparative lens has uncovered patterns and divergences that would be overlooked in isolated case studies.

The Arctic faces an increasingly uncertain future. Geopolitics, climate change, burgeoning tourism, environmental pressures and shifting social dynamics all shape how airports are used, imagined and contested. From residents and tourists to governments and industries, different actors seek different affordances from aviation infrastructure, raising the critical question: whose interests are being served? Amid these complexities, Arctic airports also embody community resilience and may enhance the visibility of remote communities within national frameworks and facilitate residents’ participation in national politics. For many, they are lifelines – enabling mobility, the flow of goods and access to services and rapid movement across vast, often impassable landscapes. While Arctic communities depend on aviation, the solution does not lie in simply building more infrastructure. Acknowledging that airport development is often driven by external economic and geopolitical interests, we argue that greater local control over transport infrastructure is needed. Ensuring that future development aligns with community needs is vital to the sustainability and resilience of Arctic communities in the long run.
